# The Schisandra-Salvia-Hawthorn herbal formulation demonstrates therapeutic efficacy in ameliorating hyperlipidemia

**DOI:** 10.3389/fphar.2026.1893568

**Published:** 2026-10-16

**Authors:** Nannan Xing, Huaidong Qu, Ying Jiang, Pengling Ge, Wei Ma

**Affiliations:** 1 College of Basic Medicine, Heilongjiang University of Chinese Medicine, Harbin, China; 2 Health Center, Harbin Normal University, Harbin, China; 3 College of Pharmacy, Heilongjiang University of Chinese Medicine, Harbin, China; 4 Experimental Teaching and Practical Training Center, Heilongjiang University of Chinese Medicine, Harbin, China

**Keywords:** disease, Hawthorn, hyperlipidemia, Salvia miltiorrhiza, Schisandra chinensis

## Abstract

**Introduction:**

This study investigates the potential efficacy and action mechanism of a herbal formulation comprising Schisandra chinensis, Salvia miltiorrhiza and Hawthorn (SSH) in the treatment of hyperlipidemia.

**Methods:**

A hyperlipidemic cell model was established by free fatty acid (FFA)-induced HepG2 cells and a hyperlipidemic mouse model was established by high-fat diet (HFD)-induced C57BL/6 mice. Cell viability of HepG2 cells was assessed by MTT assay. Serum lipid profiles (TC, TG, LDL-C, HDL-C) were quantified via the corresponding kits. Hepatic tissue pathology was evaluated via Oil Red O staining and HE staining. Serum inflammatory cytokines (TNF-α and IL-6) were measured through ELISA. The mRNA and protein expression of seven key targets (APOB, LDLR, OLR1, PPARG, PRKCA, RXRA, NCF1) identified via network pharmacology prediction, were detected using qRT-PCR and Western blot. Besides, protein levels of p-p65 and p65 were detected by Western blot. Immunofluorescence staining was used to monitor p65 subcellular distribution, and DCFH-DA-based fluorescence detection was conducted to assess ROS production.

**Results:**

In FFA-induced HepG2 cells, TC, TG, and lipid accumulation were reduced after SSH treatment. In HFD-induced mice, SSH treatment attenuated HFD-induced increases in serum TC, TG, and LDL-C levels, while elevating the level of HDL-C. It also reduced hepatic lipid accumulation, hepatic injury and expression of TNF-α and IL-6. Mechanistically, SSH formulation reversed HFD-driven dysregulation of the predicted targets: it downregulated the expression of APOB, OLR1, PRKCA, RXRA, PPARG and NCF1, and upregulated the expression of LDLR. FFA caused increases of OLR1 protein expression, PRKCA protein expression and phosphorylation of NF-κB p65 as well as ROS production in HepG2 cells, which were reversed by SSH treatment.

**Conclusion:**

The SSH formulation mitigated hyperlipidemia by restoring lipid homeostasis and suppressing inflammation. Its efficacy was mediated through a multi-target mechanism, coordinately regulating lipoprotein metabolism (APOB/LDLR), oxidative stress and inflammatory signaling (OLR1/PRKCA/NCF1), and nuclear receptor activity (PPARG/RXRA).

## 1 Introduction

Hyperlipidemia is a metabolic disorder characterized by elevated serum levels of triglycerides (TG), total cholesterol (TC), and low-density lipoprotein cholesterol (LDL-C), coupled with reduced high-density lipoprotein cholesterol (HDL-C) (Li et al., 2016; Xenoulis and Steiner, 2015). Its etiology is multifactorial, including dietary patterns (excessive intake of saturated fats and refined carbohydrates), psychology, diabetes, pathology, and genetic predisposition (Foxton, 1998; Pedro-Botet et al., 2021). Accumulating studies have linked hyperlipidemia to the development and progression of atherosclerosis, ischemic stroke, coronary artery disease, acute myocardial infarction, type 2 diabetes mellitus, and chronic kidney disease (O'Keefe and Bell, 2007; Farnier and Davignon, 1998; Osborn and Olefsky, 2012). Current first-line pharmacotherapies comprise fibrates, HMG-CoA reductase inhibitors (statins), bile acid sequestrants, niacin derivatives, and intestinal cholesterol absorption inhibitors (Illingworth, 1990; Wang et al., 2019). However, these agents are frequently limited by adverse effects: statins may induce myalgia or, rarely, rhabdomyolysis; fibrates are associated with myotoxicity and potential hepatorenal dysfunction (Wu et al., 2009; You et al., 2025). Therefore, the development of novel therapeutic interventions that combine high efficacy with enhanced safety remains a critical unmet need.

In recent years, Chinese herbal medicines have emerged as promising candidates for managing obesity and preventing metabolic disorders, owing to their favorable safety profiles and multi-target pharmacological actions (Chu et al., 2015; Panossian and Wikman, 2008). Schisandra chinensis, a cornerstone herb in Traditional Chinese Medicine (TCM), exhibits hepatoprotective, anti-inflammatory, antioxidant, antitumor, and insulin-sensitizing activities (Chiu et al., 2002; Xie and Du, 2011). Its therapeutic effects are largely attributed to dibenzocyclooctadiene lignans, collectively termed Schisandra chinensis lignans. Preclinical evidence has demonstrated that Schisandra chinensis lignans ameliorate dyslipidemia in high-fat diet (HFD)-induced hypercholesterolemia mice, notably reducing TG and TC accumulation (Sun et al., 2014; Wat et al., 2016).

Salvia miltiorrhiza is a traditional Chinese medicine that includes water-soluble components such as salyanolic acid, tanshinaldehyde and comfrey acid, which can inhibit early adipogenesis and alleviate lipid metabolism disorders (Jung et al., 2020; Chen et al., 2018). Moreover, its water-soluble constituents, including Salvianolic acids and tanshinaldehyde, suppress early-stage adipogenesis and improve systemic lipid homeostasis (Jung et al., 2020; Chen et al., 2018). Its lipophilic diterpenoid quinones (dihydrotanshinone I, tanshinone IIA/IIB, and cryptotanshinone) attenuate lipolysis and lower intracellular TG and TC stores, thereby offering protection against nonalcoholic fatty liver disease and coronary atherosclerosis (Guo et al., 2020; Pan et al., 2018).

Hawthorn (the genus Hawthorn), long used in both folk medicine and functional food applications, exhibits clinically relevant hypolipidemic, hypoglycemic and antioxidant activities (Peters et al., 2012; Niu et al., 2013). Its established cardiovascular benefits have supported its incorporation into modern nutraceutical strategies aimed at primary prevention of atherosclerotic cardiovascular disease (Gundogdu et al., 2014; Hatipoglu et al., 2015). Given the robust individual pharmacological profiles of Schisandra chinensis, Salvia miltiorrhiza and Hawthorn, the rational combination of these three botanicals represents a compelling avenue for synergistic intervention in hyperlipidemia. This approach warrants rigorous evaluation of integrated efficacy, optimal formulation ratios, and underlying molecular mechanisms. The present study explored the optimal ratio and dosage of a standardized herbal formulation comprising Schisandra chinensis, Salvia miltiorrhiza and Hawthorn(SSH). We comprehensively evaluated its anti-hyperlipidemic effects both *in vivo* and *in vitro*. Meantime, this study explored its multi-target mechanism of action, thereby establishing a pharmacological foundation for its rational development as a preventive intervention in hyperlipidemia.

## 2 Materials and methods

### 2.1 Sample preparation

Schisandra chinensis, Salvia miltiorrhiza and Hawthorn were bought from Shanghai Maclean Biochemical Technology Co., Ltd (Shanghai, China), which were ground and sieved through a 100-mesh sieve. The herbal extract was prepared via separately weighing powdered Schisandra chinensis, Salvia miltiorrhiza and Hawthorn according to the designated formulation ratios. Each batch was extracted in a decoction apparatus using distilled water at a solid-to-liquid ratio of 1:20 (w/v), followed by heating under reflux for 90 min. The mixture was centrifuged (3,000 × g, 15 min, 4 °C), and the supernatant was collected. The residue was exposed to two additional sequential extractions under identical conditions. All supernatants were pooled, concentrated under reduced pressure at ≤ 60 °C, and lyophilized to yield lyophilized extracts corresponding to the respective formulation proportions of SSH.

### 2.2 Matching principles

This study focused on Schisandra chinensis, Salvia miltiorrhiza and Hawthorn. Through uniform design and orthogonal experimental design, the formula ratios of Schisandra chinensis, Salvia miltiorrhiza, and Hawthorn were optimized. The uniform design table U_7_(7^6^) was selected based on the three-factor, seven-level experimental design. Columns 1, 2, and 3 were chosen according to the standard usage guidelines of the uniform design to construct the U_7_(7^3^) table. The three factors were defined as: Factor A = Schisandra chinensis, Factor B = Salvia miltiorrhiza, Factor C = Hawthorn. Each factor was divided into 7 equally spaced levels based on the dosage ranges recorded in the Chinese Pharmacopoeia. The complete design matrix was presented in Table 1.

**TABLE 1:** Experimental design table.

Number	Formula ratio	Number	Formula ratio
1	1:0:0	17	2:3:3
2	0:1:0	18	2:3.5:1.5
3	0:0:1	19	2.5:1:3
4	1:1:0	20	3:1:1
5	1:0:1	21	3:1:2
6	0:1:1	22	3:1:3
7	1:1.5:2	23	3:2:1
8	1:2:2	24	3:2:2
9	1:3:1	25	3:2:3
10	1:3:2	26	3:3:1
11	1:3:3	27	3:3:2
12	1.5:2.5:3.5	28	3:3:3
13	2:1:2	29	3.5:3:2.5
14	2:2:3	30	3:2:0
15	2:3:1	31	3:0:1
16	2:3:2	32	0:2:1

### 2.3 Cell culture and treatment

The human hepatoma (HepG2) cell line was bought from the Cell Bank of the Chinese Academy of Sciences (Shanghai, China). The cells were cultured in Dulbecco’s modified Eagle’s medium (DMEM, Gibco, Grand Island, NY, United States) containing 10% FBS (Gibco) and 1% penicillin-streptomycin (Gibco). Cells were incubated at 37 °C in an atmosphere containing 5% CO_2_. HepG2 cells were used between passages 5 and 10 for following experiments. Moreover, HepG2 cells were tested for *mycoplasma* contamination using a *Mycoplasma* Detection Kit-Quick Test (iNtRON, Shanghai, China), and all experiments were performed with mycoplasma-free cells. The HepG2 cell line was authenticated by short tandem repeat (STR) profiling within the last 6 months. All cell-based experiments were performed using at least three independent replicates. Free fatty acid (FFA) stock solution was prepared by dissolving equimolar amounts of oleic acid and palmitic acid in 0.1 mmol/L sodium hydroxide (NaOH) solution, followed by incubation in a water bath at 70 °C for 20 min to ensure complete saponification and solubilization. The resulting solution was adjusted to a final concentration of 10 mmol/L with 10% (w/v) bovine serum albumin (BSA) in phosphate-buffered saline (PBS), vortexed gently, and filtered through a 0.22-μm sterile filter. The working solution was aliquoted and stored at 4 °C, protected from light, and used within 72 h.

For experimental assays, HepG2 cells were seeded into 12-well plates at a density of 1 × 10^5^ cells per well. Following a 24-h adherence and recovery period, cells were exposed to 1 mmol/L FFA for an additional 24 h, establishing the cell model of hyperlipidemia. To explore the optimum proportion of SSH formulation against FFA-induced lipotoxicity, HepG2 cells in the model group were treated with SSH formulation (1:2:3 [D1], 2:4:6 [D2], 3: 6:2 [D3], 4:1:5 [D4], 5:3:2 [D5], 6:5:4 [D6], 1:1:1 [D7]) for 24 h.

The optimal formulation was selected based on a composite score that integrated two equally weighted outcomes: (a) cell viability (MTT assay, reflecting safety) and (b) intracellular TG content (reflecting efficacy), both evaluated in HepG2 cells. The composite score was calculated as: Score = (Cell viability/the control group cell viability) × 0.5 + (1 - Lipid content/the model group lipid content) × 0.5. D5 achieved the highest composite score (0.67) among all seven formulations, showing the most favorable balance between safety and efficacy (highest cell viability with lowest lipid accumulation), leading to its selection as the optimal formulation. The scores for the seven formulations (D1-D7) were presented in Supplementary Table S1.

### 2.4 Animals

C57BL/6 mice (male, 6 weeks of age) were bought from Beijing Vital River Laboratory Animal Technology Co., Ltd. (Beijing, China), which were allowed to acclimate to their surroundings for 1 week. All mice were housed in an air-conditioned room (temperature 22 °C ± 2 °C, humidity of 50%–60%, 12 h light/dark cycle). All experiments were performed in accordance with the ARRIVE 2.0 guidelines and complying the guidelines approved by the Animal Protection and Medical Ethics Committee of Heilongjiang University of Chinese Medicine (2026052929).

### 2.5 Experimental protocols *in vivo*

Mice were respectively fed with control feed (an AIN-93M control diet) and HFD for 4 weeks. During the modeling process, the concentrations of TG and TC in the plasma of each mouse were measured by taking blood from the tail tip. When the concentrations of TG and TC in the plasma of the model group mice were twice that of the control group mice, it suggested successful establishment of the model. Overall, mice were randomly divided into following groups (n = 10 in each group): control, HFD, HFD + Schisandra chinensis (300 mg/kg), HFD + Salvia miltiorrhiza (200 mg/kg), HFD + Hawthorn (100 mg/kg), HFD + SSH formulation (600 mg/kg), HFD + SSH formulation (1200 mg/kg), HFD + SSH formulation (1800 mg/kg) and HFD + positive (Simvastatin, 10 mg/kg). The single-herb doses (Schisandra chinensis: 300 mg/kg; Salvia miltiorrhiza: 200 mg/kg; Hawthorn: 100 mg/kg) were selected to match the proportional contribution of each herb within the mid-dose SSH formulation (600 mg/kg) according to the D5 ratio (5:3:2), thereby enabling a valid comparison between the combination therapy and each monotherapy. Specifically, these values correspond to the lyophilized extract weight, and the crude-herb equivalent doses (900, 540, and 360 mg/kg, respectively) are provided in the figure legends for reference. The SSH formulation group (600, 1200, and 1800 mg/kg) refers to the total lyophilized extract weight of the co-formulated SSH combination.

During weeks 5–10, mice in the control group were maintained on the AIN-93M standard control diet, while those in the HFD group received a HFD. The other groups were fed an HFD supplemented with above mentioned traditional Chinese Medicines. The formulaiton included Schisandra chinensis, Salvia miltiorrhiza and Hawthorn. All diets were provided by Beijing Keao Xieli Feed Co., Ltd. (Beijing, China), and dietary compositions were exhibited in Table 2. At the end of the experiment, mice were fasted for 16 h. Next day, mice were weighed and anesthetized by intraperitoneal injection of barbital and blood samples were collected by retro-orbital bleeding. Serum samples were collected by centrifugation at 4 °C and 4,000 r/min for 10 min, and then stored at −80 °C for further analysis. Mice were weighed immediately and then mouse liver tissues were collected and stored at −80 °C for further analysis.

**TABLE 2:** The composition of diets in this study.

Ingredient (g)	Control diet	High-fat diet
Cornstarch	465.7	235.7
Casein	140	110
Dextrinized cornstarch	155	155
Sucrose	100	100
Soybean oil	40	40
Choline bitartrate	2.5	2.5
Fiber^a^	50	50
Mineral mix^b^	35	35
Vitamin mix^c^	10	10
L-Cysteine	1.8	1.8
Lard	0	150
Cholesterol	0	10
Yolk	0	100
NO powder	0	0
Total	1000	1000

^a^ Solka-Floc cellulose.

^b^ AIN-93, mineral mix.

^c^ AIN-93, vitamin mix.

### 2.6 3-(4,5-dimethylthiazol-2-yl)-2,5-diphenyltetrazolium bromide (MTT) *assay*

The viability of HepG2 cells was assessed by MTT assay. Cells were seeded in 96-well plates (1 × 10^4^ cells/well) and treated for 24 h. Thereafter, 20 µL of MTT solution (5 mg/mL in PBS) was added to each well, followed by 4 h incubation at 37 °C. The formazan crystals formed were solubilized by removing the supernatant and adding 150 µL dimethyl sulfoxide (DMSO) per well. Plates were gently shaken in the dark for 15 min to ensure complete dissolution, and absorbance was measured at 490 nm using a Bio-Rad microplate reader (Hercules, CA, United States).

### 2.7 Oil Red O (ORO) staining

For *in vitro* assessment, HepG2 cells were rinsed with PBS, fixed with 4% neutral-buffered formalin for 30 min at room temperature, and then stained with 0.5% Oil Red O solution (Servicebio, Wuhan, China) for 20 min. Excess dye was removed by differentiation in 75% ethanol for 2 min, and images were acquired using an inverted light microscope.

For *in vivo* evaluation, frozen liver sections (8 µm thick) were prepared from snap-frozen tissues and stained with 0.5% ORO solution for 30 min. Sections were differentiated in 75% ethanol, counterstained with hematoxylin, dehydrated through graded ethanol, cleared in xylene, and mounted with neutral balsam. Imaging was performed under identical microscope settings.

### 2.8 Liver index

The liver was rinsed in pre-cooled normal saline and sucked dry with filter paper. The liver index was calculated by the following equation: Liver index = liver weight/body weight × 100%.

### 2.9 Intracellular TC and TG quantification

HepG2 cells (1 × 10^5^/mL) were seeded in 6-well plates with 2 mL per well and cultured in an incubator at 37 °C. Then cells were washed with cold PBS and lysed. The intracellular TG mass was quantified spectrophotometrically at 510 nm using a TG test kit (Nanjing Jiancheng, China), and TC mass was quantified spectrophotometrically at 490 nm using a TC test kit (APPLYGEN, Beijing, China).

### 2.10 Serum lipids analysis in mice

The concentrations of TC, TG, LDL-C, and HDL-C in mouse serum were determined using commercial assay kits (Nanjing Jiancheng). Serum contents of TNF-α and IL-6 were determined using CUSABIO ELISA kits (Wuhan, China).

### 2.11 Hematoxylin and eosin (HE) staining

HE staining was performed to evaluate the general morphology of mouse livers. The isolated livers were fixed with 4% paraformaldehyde for more than 24 h and the fixed tissues were dehydrated with a gradient with alcohol. Following paraffin embedding the dehydrated tissues, tissues were cut into sections (3 µm thick). Slices were dewaxed with xylene and stained with hematoxylin eosin (Servicebio). After dehydration with conventional ethanol, the slices were sealed. Finally, the samples were observed with a light microscopy (Olympus, Japan).

### 2.12 Pharmacological analysis

The chemical components of Schisandra chinensis, Salvia miltiorrhiza, and Hawthorn were retrieved through the TCMSP (https://tcmsp-e.com/) and ETCM databases. The screening criteria were oral bioavailability (OB) ≥ 30% and drug similarity (DL) ≥ 0.18, and the candidate active compounds were obtained. The protein targets corresponding to the active compounds were extracted from the TCMSP database. The gene ontology (GO) enrichment analysis (including biological process, cellular component and molecular function) and Kyoto Encyclopedia of Genes and Genomes (KEGG) pathway enrichment analysis were conducted using R version 4.3.1 and the clusterProfiler 4.8.1 package. The significance threshold was set at *P* < 0.05 and FDR <0.05. The top-ranked enriched items were visualized using bubble charts. The gene set of the Lipid_and_atherosclerosis pathway in KEGG analysis was extracted and plotted with the gene set from the Lipidmaps database (funded by the National Institutes of Health in 2003 for lipid metabolism pathway research). The intersection of the genes in the two sets was the candidate therapeutic targets.

### 2.13 Quantitative real-time polymerase chain reaction (qRT-PCR)

Total RNAs were extracted from mouse liver tissues through a commercial RNA extraction kit (Servicebio). RNA concentration and purity were measured spectrophotometrically with the NanoDrop 2000c system (Thermo Fisher Scientific, Waltham, MA, United States). For reverse transcription, 2 µg of each RNA sample was converted into complementary DNA with a PrimeScript™ RT reagent Kit (Takara, Beijing, China). qRT-PCR was performed on a CFX96 Real-Time System (Bio-Rad) using SYBR® Premix Ex Taq™ II (Takara) under the following cycling conditions: denaturation at 95 °C for 15 s, primer annealing at 61 °C for 30 s, and extension at 61 °C for 30 s. Relative mRNA expression of APOB (apolipoprotein B gene), OLR1 (encoding Lectin-like oxidized low-density lipoprotein receptor 1), PRKCA (Protein kinase C-alpha), RXRA (retinoid X receptor alpha), PPARG (gene symbol of PPARγ protein), NCF1 (neutrophil cytosolic factor 1) and LDLR (low-density lipoprotein receptor) was calculated via the 2^−ΔΔct^ method, which was normalized to GAPDH. Primer sequences were shown in Table 3.

**TABLE 3:** Primer sequences for quantitative real-time polymerase chain reaction (qRT-PCR).

Genes	Primer	Sequences (5’∼3’)
APOB	Forward	GCT​CAA​CTC​AGG​TTA​CCG​TGA
	Reverse	AGG​GTG​TAC​TGG​CAA​GTT​TGG
OLR1	Forward	CAA​GAT​GAA​GCC​TGC​GAA​TGA
	Reverse	ACC​TGG​CGT​AAT​TGT​GTC​CAC
PRKCA	Forward	TGA​AAG​ACC​ACA​AAT​TCA​TCG​CC
	Reverse	ACG​AAC​TCA​TGG​CAC​CTC​TTA​T
RXRA	Forward	ATG​GAC​ACC​AAA​CAT​TTC​CTG​C
	Reverse	CTC​GAC​CCG​TTG​GAG​AGT​T
PPARG	Forward	GGA​AGA​CCA​CTC​GCA​TTC​CTT
	Reverse	GTA​ATC​AGC​AAC​CAT​TGG​GTC​A
NCF1	Forward	ACA​CCT​TCA​TTC​GCC​ATA​TTG​C
	Reverse	CCT​GCC​ACT​TAA​CCA​GGA​ACA
LDLR	Forward	TCA​GAC​GAA​CAA​GGC​TGT​CC
	Reverse	CCA​TCT​AGG​CAA​TCT​CGG​TCT​C
GAPDH	Forward	TGG​CCT​TCC​GTG​TTC​CTA​C
	Reverse	GAG​TTG​CTG​TTG​AAG​TCG​CA

### 2.14 Western blot

Total protein was extracted from liver tissues using RIPA lysis buffer (Beyotime, Shanghai, China) supplemented with protease and phosphatase inhibitors. Protein concentration was determined using the BCA Protein Assay Kit (Solarbio, Beijing, China). Equal amounts (20 µg per lane) were resolved by SDS-PAGE on 10% polyacrylamide gels (Solarbio). Electrophoretically separated proteins were transferred onto polyvinylidene difluoride (PVDF) membranes (Pall Corporation, Port Washington, NY, United States). Membranes were blocked with 5% non-fat dry milk for 1 h, followed by overnight incubation at 4 °C with appropriate primary antibodies including anti-APOB (1:1000, #10067, Cell Signaling Technology, Danvers, Massachusetts, United States), anti-OLR1 (1:1000, #57946, Cell Signaling Technology), anti-PRKCA (1:1000, #59754, Cell Signaling Technology), anti-RXRA (1:1000, #3085, Cell Signaling Technology), anti-PPARG (1:1000, #2435, Cell Signaling Technology), anti-NCF1 (1:1000, #63290, Cell Signaling Technology), anti-p-NF-κB p65 (1:1000, #3033, Cell Signaling Technology), anti-NF-κB p65 (1:1000, #8242, Cell Signaling Technology) and anti-LDLR (1:1000, HY-P83717, MedChemExpress, Shanghai, China). Next day, membranes were probed with a horseradish peroxidase (HRP)-conjugated goat anti-rabbit secondary antibody (1:5,000, SanYing, Beijing, China) for 1 h. Immunoreactive bands were visualized using an enhanced chemiluminescence (ECL) substrate (Thermo Fisher Scientific), and band intensities were quantified through ImageJ software (National Institutes of Health, United States).

### 2.15 Immunofluorescence assay

HepG2 cells were seeded on glass coverslips in 24-well plates and allowed to adhere overnight. Cells were fixed with ice-cold methanol for 10 min at 4 °C. After washing with PBS, cells were permeabilized with 0.2% Triton X-100 in PBS for 10 min, followed by blocking with 5% BSA for 1 h at room temperature. HepG2 cells were then incubated with the primary antibody against NF-κB p65 (1:500, #8242, Cell Signaling Technology) overnight at 4 °C. After washing three times with PBS, cells were incubated with an anti-rabbit secondary antibody for 1 h at room temperature in the dark. Nuclei were counterstained with 4′,6-diamidino-2-phenylindole (DAPI) for 5 min. After final washes, coverslips were mounted onto glass slides using an anti-fade mounting medium. Images were captured using a fluorescence microscope (EVOS FL Auto 2 imaging system; Thermo Fisher Scientific).

### 2.16 Detection of reactive oxygen species (ROS)

HepG2 cells were seeded in 24-well plates. After treatment, the culture medium was removed, and cells were washed twice with serum-free medium. Cells were then incubated with 200 μL of 10 μM DCFH-DA (Beyotime) in serum-free medium for 20 min in the dark. After incubation, cells were washed twice with PBS to remove excess probe. The intracellular ROS level was assessed by measuring fluorescence intensity using a Leica fluorescence microscope with an excitation wavelength of 488 nm and an emission wavelength of 525 nm.

### 2.17 Statistical analysis

All statistical analysis was conducted using GraphPad Prism 6 (GraphPad Software Inc., San Diego, United States) and data were displayed as mean ± standard deviation (SD). For comparisons between two groups, unpaired two-tailed Student’s t-test was used. For comparisons among three or more groups, one-way analysis of variance (ANOVA) was applied, followed by Tukey’s multiple comparisons test as a *post hoc* procedure. A value of *P* < 0.05 indicated a statistically significant difference.

## 3 Results

### 3.1 The optimum proportion of SSH formulation SSH formulation protects against FFA-induced lipotoxicity in HepG2 cells

We firstly examined the effects of SSH (D1-D7) on HepG2 cell viability and found that none of the treatments caused significant cell death or reduced viability below 85% compared to the control group (Figure 1A). Then FFA was used to establish a hyperlipidemic cell model using HepG2 cells. Following FFA exposure, the TG concentration was markedly increased (*P* < 0.001; Figure 1B), confirming successful induction of a lipid-overload state. Subsequently, FFA-primed HepG2 cells were exposed to the SSH formulation for 24 h. We discovered that SSH formulation (from D1 to D5) reduced the cellular TG level in a dose-dependent manner, with most significant reduction observed at D5 in FFA-induced HepG2 cells (*P* < 0.01, Figure 1B). ORO staining also showed consistent result in reduction of lipid droplets in FFA-induced HepG2 cells (Figure 1C). Based on these results and the uniform design optimization, the D5 formulation was identified as the optimal ratio and was therefore used as the standard SSH formulation for all subsequent *in vitro* and *in vivo* experiments.

**FIGURE 1:**
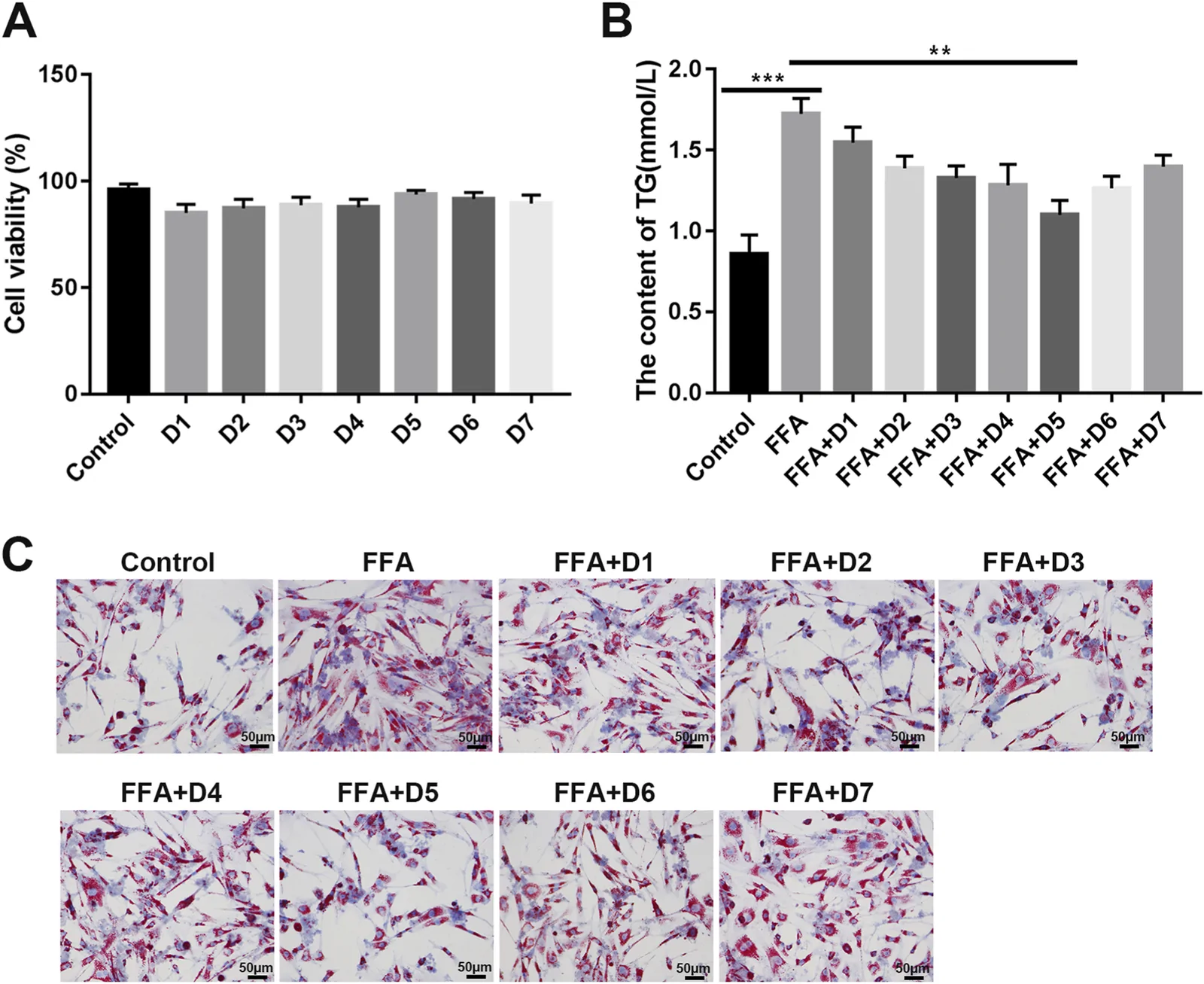
The optimum proportion of SSH formulation protects against FFA-induced lipotoxicity in HepG2 cells. **(A)** The viability of HepG2 cells was determined using MTT assay. **(B)** The level of triglyceride (TG) in HepG2 cells was shown. **(C)** Oil red O-staining was employed to assess lipid accumulation of HepG2 cells in each group. ^**^ *P* < 0.01, ^***^ *P* < 0.001.

### 3.2 SSH formulation represses lipid accumulation in FFA-induced HepG2 cells

Afterwards, we investigated the effects of different doses of SSH formulation (optimal proportion) on HepG2 cells. We found that SSH formulation dose was safe and effective within 500–1000 μg/mL. The range of 500–1000 μg/mL was identified because, within this range, the viability of HepG2 cells was consistently ≥85% (Figure 2A). However, the concentration of 1500 μg/mL resulted in cell viability below 80% (77.26%, Figure 2A), indicating potential cytotoxicity. Meanwhile, 1500 μg/mL SSH formulation caused a lower increase in cell viability compared to 1000 μg/mL SSH formulation in FFA-induced HepG2 cells (Figure 2B). More importantly, lipophilic ORO staining showed that SSH formulation treatment (500 and 1000 μg/mL) alleviated hepatic steatosis in FFA-induced HepG2 cells (Figure 2C). Meanwhile, SSH formulation treatment reduced levels of TG and TC in FFA-induced HepG2 cells (*P* < 0.01, Figures 2D,E). These results suggested that the lipid-lowering effects of 500 and 1000 μg/mL SSH formulation on FFA-induced HepG2 cells were statistically significant. Thus, we used 500 and 1000 μg/mL SSH formulation to perform following experiments.

**FIGURE 2:**
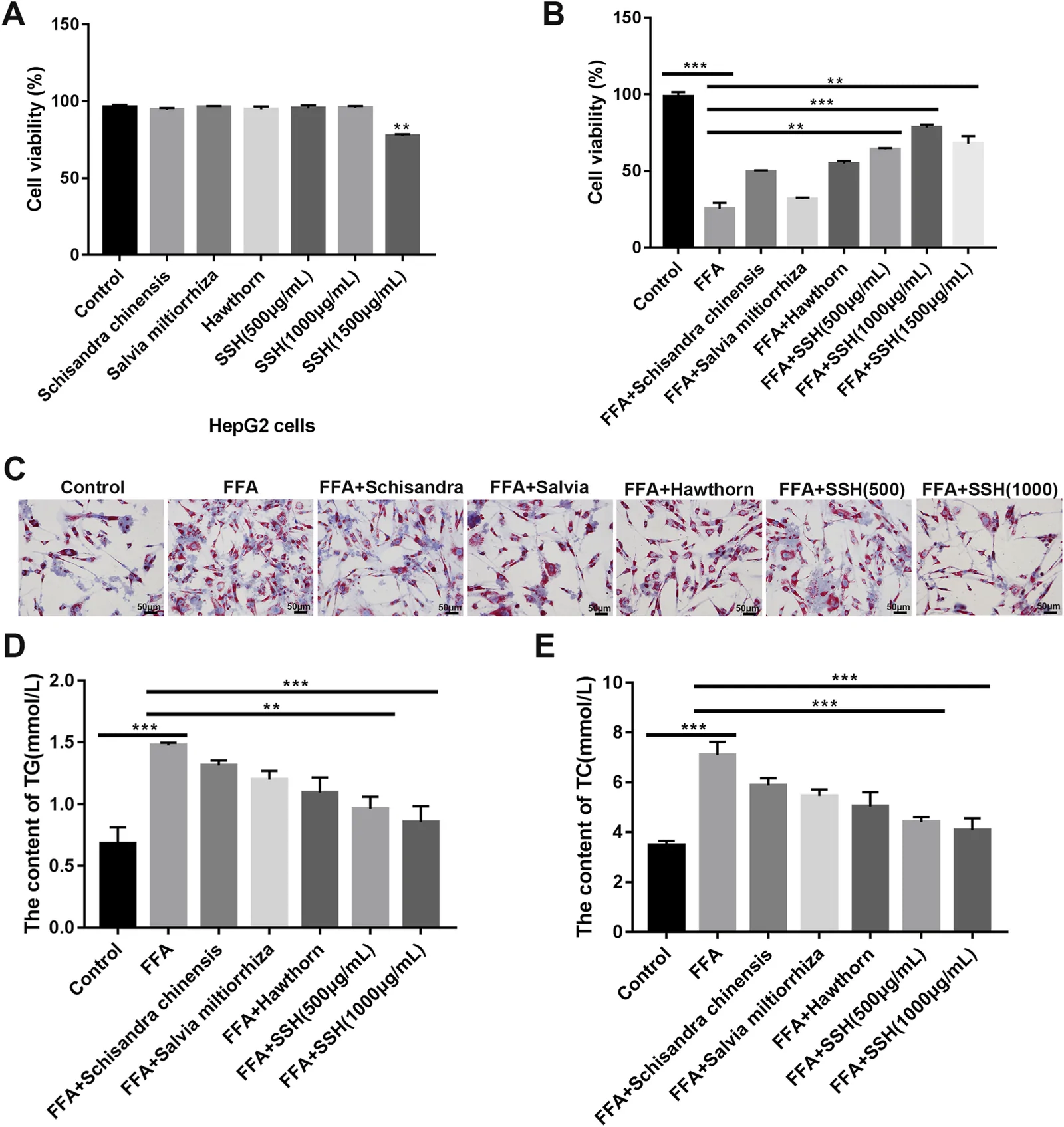
SSH formulation represses lipid accumulation in FFA-induced HepG2 cells. **(A)** Cell viability of HepG2 cells was detected through MTT assay. Schisandra chinensis extract: 750 μg/mL (50% of 1500 μg/mL, corresponding to the 5:3:2 ratio); Salvia miltiorrhiza extract: 450 μg/mL (30% of 1500 μg/mL); Hawthorn extract: 300 μg/mL (20% of 1500 μg/mL). **(B)** Cell viability of HepG2 cells was detected through MTT assay. **(C)** Oil red O-staining was used to assess lipid accumulation of HepG2 cells in each group. **(D,E)** The levels of triglyceride (TG) and total cholesterol (TC) in HepG2 cells were shown. ^**^ *P* < 0.01, ^***^ *P* < 0.001.

### 3.3 SSH formulation alleviates hyperlipidemia in HFD-induced mouse model

We then explored the effects of SSH formulation on HFD-fed mice. As displayed in Figures 3A,B, significant increases in body weight and liver index were observed in the HFD group mice compared to the control group mice (*P* < 0.001), while administration with SSH formulation reduced mouse body weight and liver index (*P* < 0.01). Serum lipid levels were also measured, and increased levels of TC, TG, LDL-C as well as decreased HDL-C were observed in the HFD-fed group, suggesting that hyperlipidemia model animals were successfully established. After administration of SSH formulation, TG (*P* < 0.05, Figure 3C), TC (*P* < 0.05, Figure 3D), and LDL-C (*P* < 0.01, Figure 3E) levels in mouse serum were evidently decreased and the HDL-C(*P* < 0.01, Figure 3F) level in serum was increased.

**FIGURE 3:**
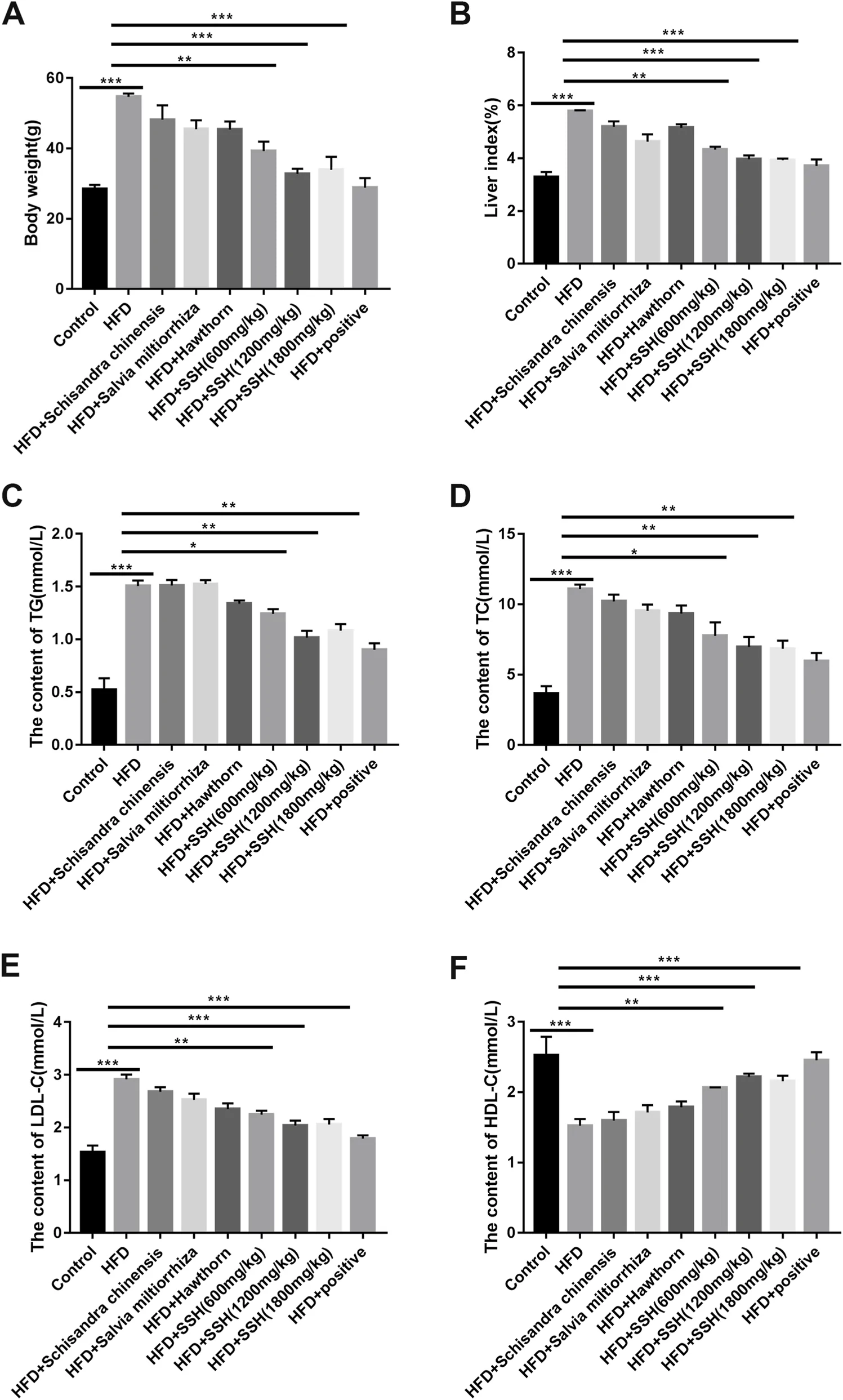
SSH formulation alleviates hyperlipidemia in HFD-induced mouse model. **(A)** The effect of SSH formulation on mouse body weight. **(B)** The effect of SSH formulation on mouse liver index. **(C,D)** The levels of triglyceride (TG) and total cholesterol (TC) in serum of mice were shown. **(E,F)** The levels of low density lipoprotein cholesterol (LDL-C) and high density lipoprotein cholesterol (HDL-C) in serum of mice were shown. The SSH formulation was administered at 1800 mg/kg/day (total extract weight). For single-herb treatment groups, the doses were: Schisandra chinensis extract: 900 mg/kg/day; Salvia miltiorrhiza extract: 540 mg/kg/day; Hawthorn extract: 360 mg/kg/day. ^*^ *P* < 0.05, ^**^ *P* < 0.01, ^***^ *P* < 0.001.

### 3.4 Inflammation and histopathological analysis

As shown in Figure 4A, serum concentrations of TNF-α and IL-6 were higher in HFD-fed mice than those of the control diet mice, while they were significantly reduced in the SSH formulation treatment groups (*P* < 0.001). Furthermore, histopathological examination revealed that hepatocytes surrounding blood vessels in the control group exhibited abundant cytoplasm and no signs of steatosis (Figure 4B). In contrast, HFD-fed mice displayed prominent perivascular micro- and macrovesicular lipid vacuoles of heterogeneous size (Figure 4B). Treatment with the SSH formulation dose-dependently ameliorated hepatic structural disruption, attenuated hepatocellular steatosis, and reduced lipid vacuole accumulation (Figure 4B). ORO staining further confirmed the absence of hepatic lipid deposition in control mice, whereas HFD-fed mice exhibited extensive orange-red staining indicative of severe intrahepatic lipid accumulation (Figure 4C). Notably, SSH formulation treatment significantly decreased hepatic lipid deposition in a dose-dependent manner (Figure 4C).

**FIGURE 4:**
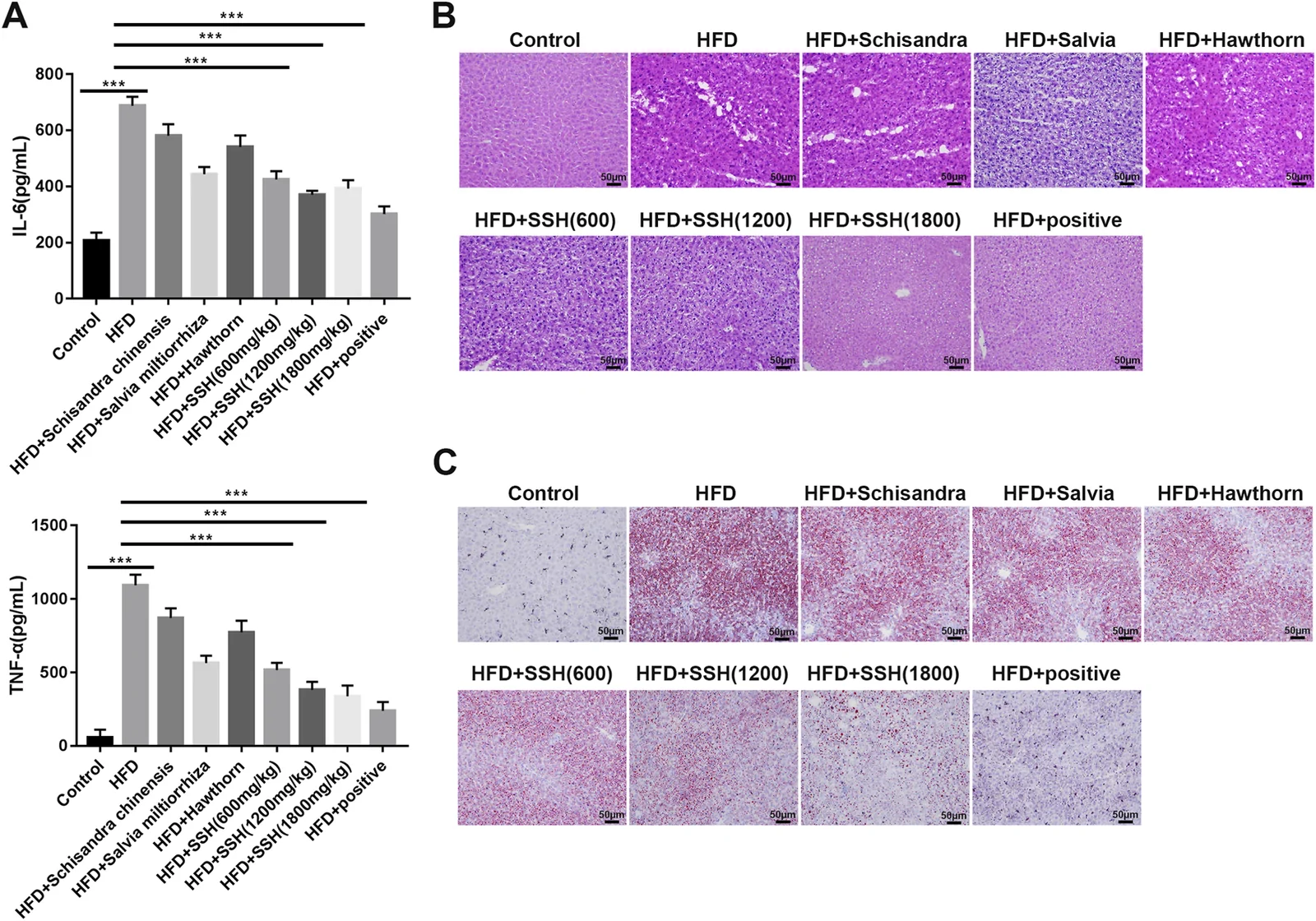
Inflammation and histopathological analysis. **(A)** Levels of TNF-α and IL-6 in serum of mice were assessed using commercial test kits. **(B)** Representative images of hematoxylin-and eosin-stained sections of mouse liver tissues. **(C)** Oil red O-staining of mouse livers in each group. ^***^ *P* < 0.001.

### 3.5 Identification of key targets and pathways underlying SSH formulation–mediated protection against hyperlipidemia via network pharmacology

To elucidate the molecular mechanisms by which the SSH formulation exerted its anti-hyperlipidemic effects, we performed integrated Gene Ontology (GO) and Kyoto Encyclopedia of Genes and Genomes (KEGG) enrichment analyses on predicted target genes of SSH formulation (Schisandra chinensis, Salvia miltiorrhiza, and Hawthorn). GO enrichment results were presented in Figure 5A. KEGG pathway mapping revealed that target genes were most prominently associated with lipid and atherosclerosis-related signaling (Figure 5B). Afterwards, by combining the dataset obtained from the LipidMap database with the lipid metabolism-related pathways (Lipid_and_atherosclerosis), the seven intersection genes were taken in the Venn diagram (Figure 5C).

**FIGURE 5:**
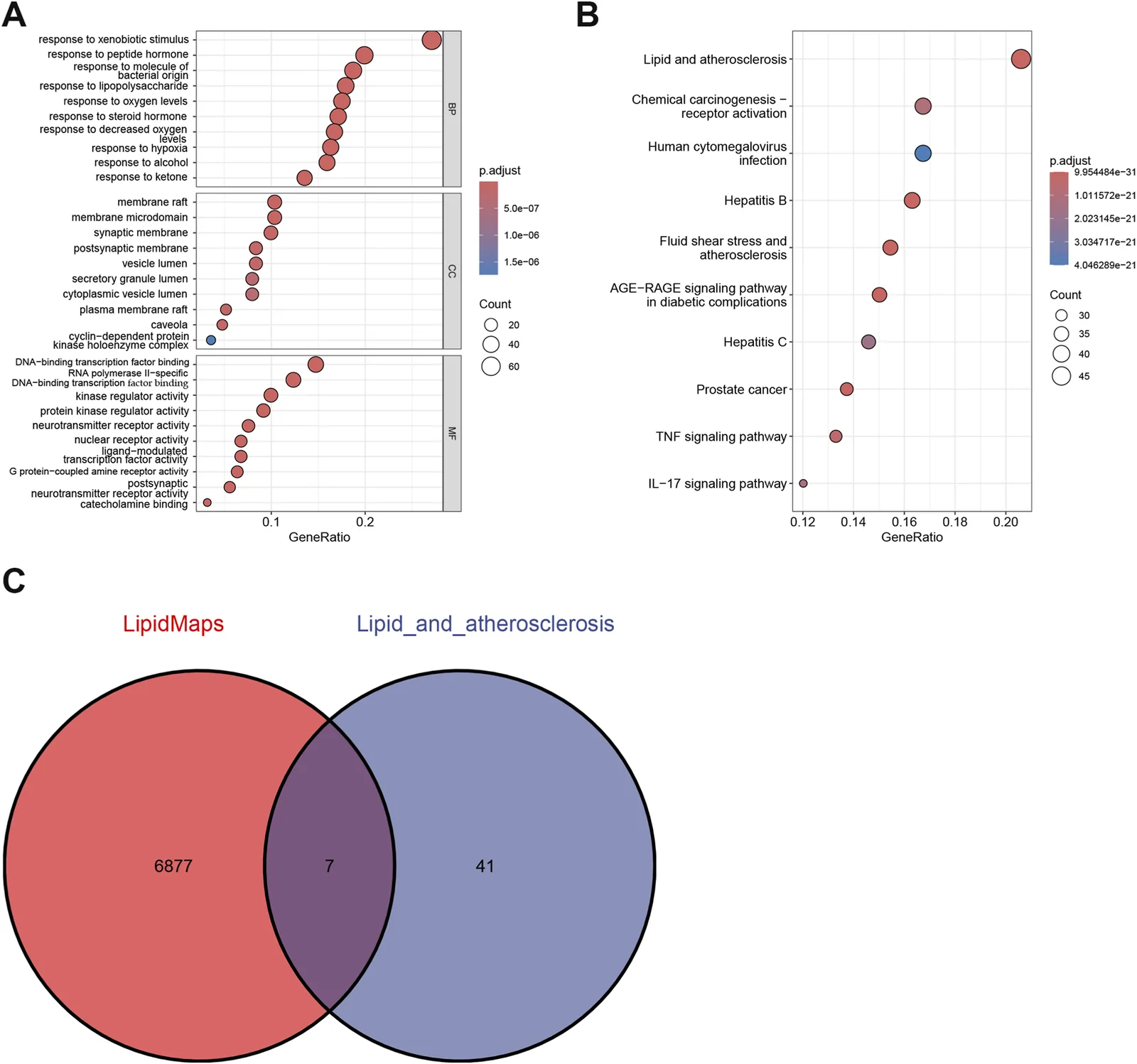
Predicting the key targets and pathways of SSH formulation in treating hyperlipidemia through network pharmacology. **(A,B)** Based on the components of SSH formulation (Schisandra chinensis, Salvia miltiorrhiza, and Hawthorn), the corresponding target genes were used for GO and KEGG analysis. **(C)** By combining the dataset obtained from the LipidMap database with the lipid metabolism-related pathways (Lipid_and_atherosclerosis), the intersection was taken to obtain the Venn diagram.

### 3.6 Expression validation of intersection genes

Then we validated the mRNA and protein expression levels of the seven intersection genes identified above using qRT-PCR and Western blotting, respectively. As shown in Figures 6A–G, hepatic mRNA expression of APOB, OLR1, PRKCA, RXRA, PPARG, and NCF1 was significantly upregulated in HFD-fed mice relative to controls (*P* < 0.001), whereas LDLR transcription was markedly suppressed (*P* < 0.001).). Importantly, SSH formulation treatment effectively reversed these HFD-induced transcriptional alterations for all seven genes (*P* < 0.05, Figures 6A–G). Consistently, protein expression levels of APOB, OLR1, PRKCA, RXRA, PPARG and NCF1 in mouse liver tissues were dramatically elevated after HFD induction, and LDLR expression was markedly decreased after HFD induction (*P* < 0.001, Figures 6H–O). Likewise, SSH formulation counteracted these HFD-driven alterations across all seven genes (*P* < 0.05, Figures 6H–O).

**FIGURE 6:**
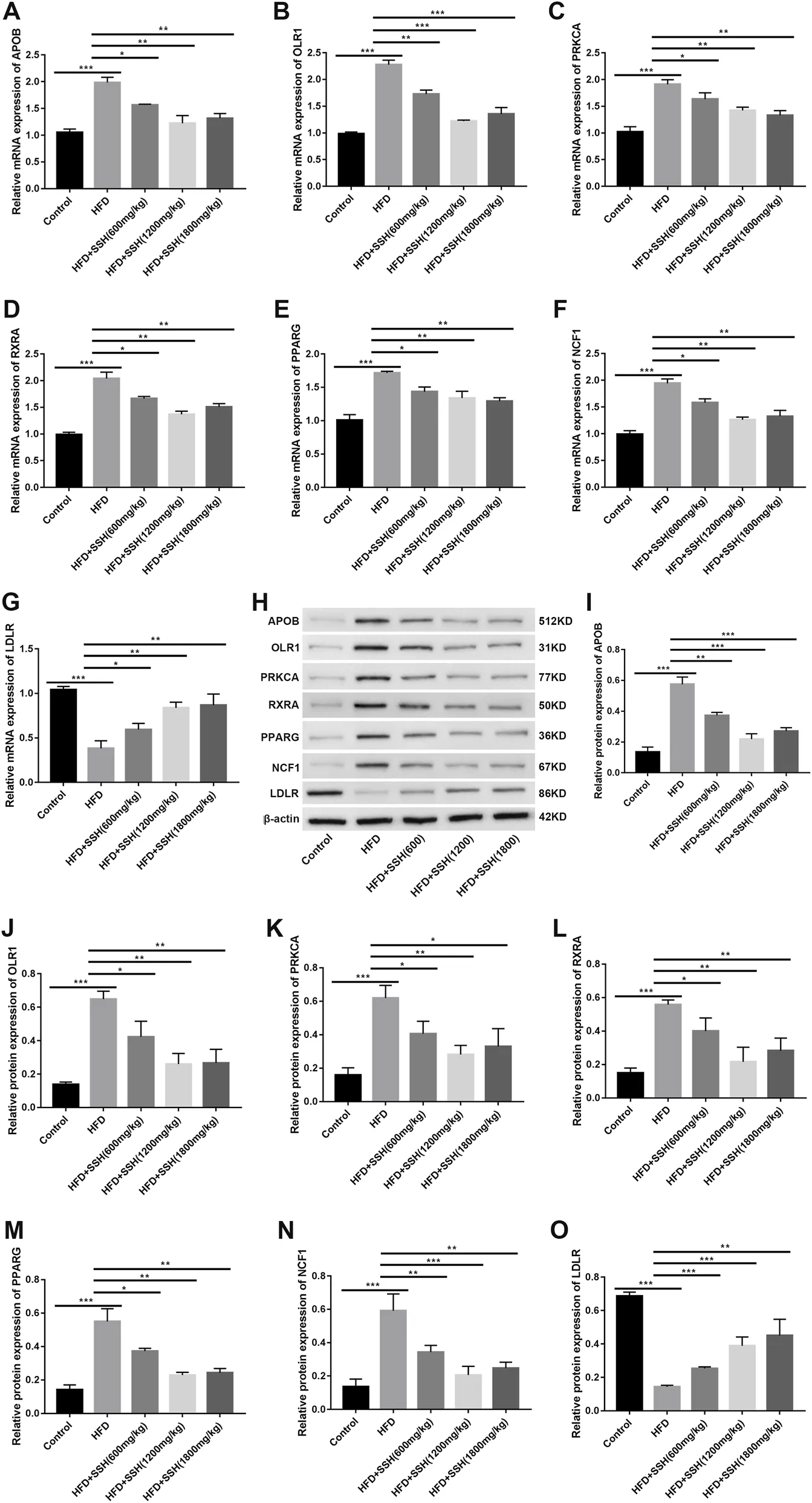
Expression validation of intersection genes. **(A–C)** Quantitative real-time polymerase chain reaction (qRT-PCR) was used to detect relative mRNA expression of APOB, OLR1 and PRKCA. **(D–G)** QRT-PCR was used to detect relative mRNA expression of RXRA, PPARG, NCF1 and LDLR. **(H–O)** Protein expression levels of APOB, OLR1, PRKCA, RXRA, PPARG, NCF1 and LDLR were detected by Western blot and quantified. ^*^ *P* < 0.05, ^**^ *P* < 0.01, ^***^ *P* < 0.001.

### 3.7 SSH exerts its effects primarily through the OLR1/PRKCA/ROS/NF-κB signaling pathway

Based on the results presented in Figure 6, we next validated the mechanism of SSH in FFA-induced HepG2 cells. It was found that FFA resulted in increases of OLR1 expression, PRKCA expression and phosphorylation of NF-κB p65 (*P* < 0.001, Figure 7A), which were reversed by SSH treatment (*P* < 0.01, Figure 7A). To assess NF-κB pathway activation, we conducted immunofluorescence staining to monitor p65 subcellular distribution. In control cells, NF-κB p65 was predominantly localized in the cytoplasm and FFA induction caused a marked translocation of NF-κB p65 from the cytoplasm to the nucleus (Figure 7B). SSH treatment inhibited this nuclear translocation, as evidenced by the restored cytoplasmic localization of NF-κB p65 (Figure 7B). Moreover, DCFH-DA-based fluorescence detection demonstrated that FFA induction increased ROS production compared to the control group and SSH treatment attenuated FFA-caused ROS increase (Figure 7C).

**FIGURE 7:**
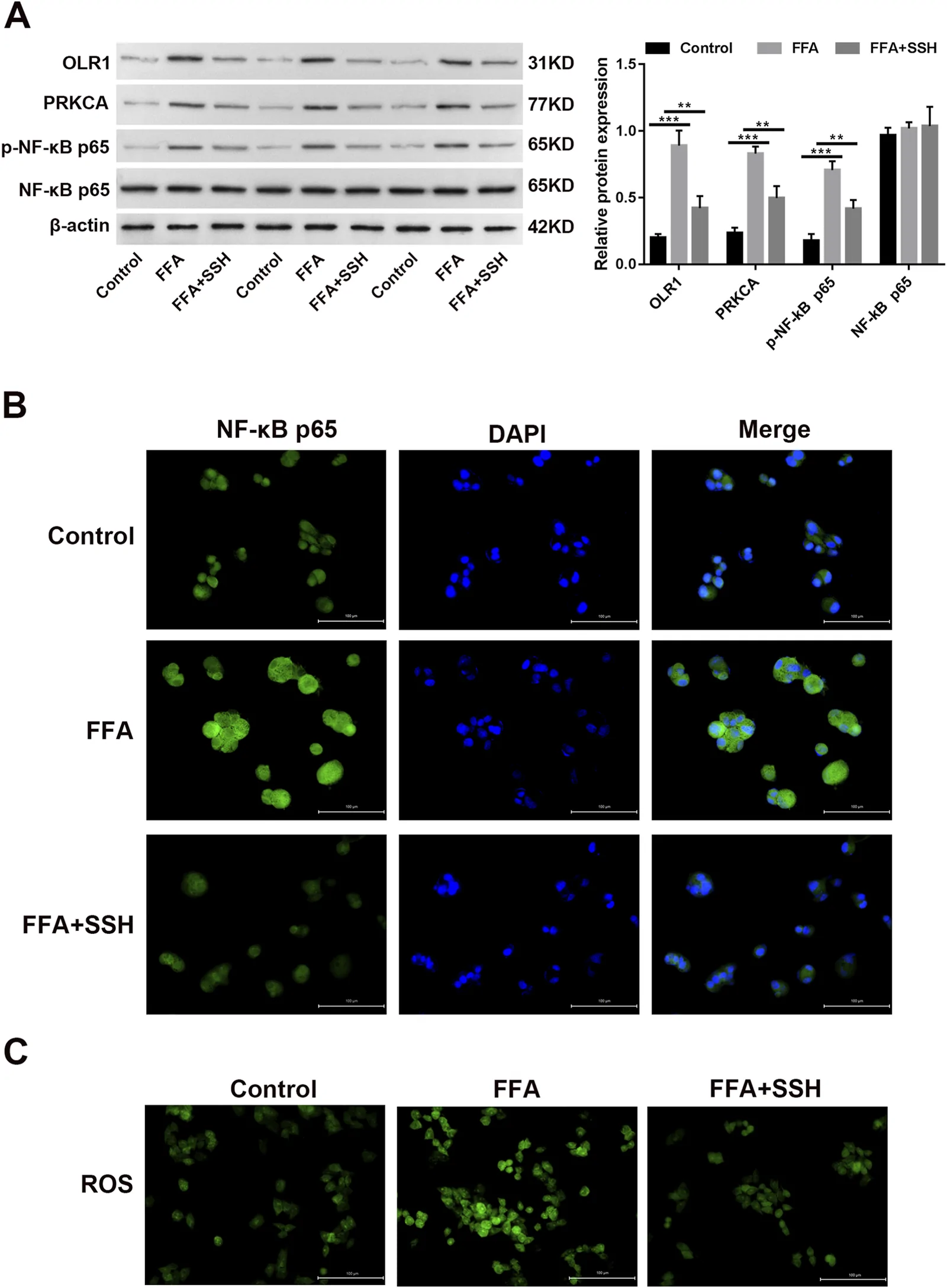
SSH exerts its effects primarily through the OLR1/PRKCA/ROS/NF-κB signaling pathway. **(A)** Protein expression levels of OLR1, PRKCA, p-p65 and p65 were detected by Western blot. **(B)** Immunofluorescence staining was used to monitor p65 subcellular distribution. **(C)** DCFH-DA-based fluorescence detection was performed to assess ROS production. ^**^ *P* < 0.01, ^***^ *P* < 0.001.

## 4 Discussion

Hyperlipidemia is a well-established risk factor for multiple chronic diseases and is associated with reduced longevity (Hatipoglu et al., 2015; Assy et al., 2000). Given the substantial adverse effects of current pharmacotherapies for hyperlipidemia, traditional Chinese herbs represent a promising alternative strategy for lipid management (Wierzbicki et al., 2003; Fabryova and Nemcova, 2020). In the present study, we innovatively proposed a novel herbal formulation SSH comprising Schisandra chinensis, Salvia miltiorrhiza and Hawthorn, optimizing its component ratio and evaluating its anti-hyperlipidemic activity. Accumulating evidence has indicated that hyperlipidemia frequently induces hepatic metabolic dysfunction, ectopic lipid accumulation, and the elevated TG level (Zhang et al., 2019; Miller, 2000). In line with prior work, we discovered increased the cellular TG level and lipid accumulation in FFA-induced HepG2 cells. Although derived from hepatocellular carcinoma, HepG2 cells retain key metabolic functions and gene expression patterns of primary human hepatocytes, making them a widely accepted model for studying hepatic lipid metabolism *in vitro* (Sefried et al., 2018; Tanaka-Yachi et al., 2022). Notably, treatment with the SSH formulation significantly reduced intracellular TG accumulation and lipid droplet area in FFA-induced HepG2 cells, with maximal efficacy observed at the D5 formulation ratio. Subsequent dose response experiments using this optimized ratio demonstrated that SSH treatment dose-dependently decreased lipid droplets, as well as cellular TG and TC levels. To our knowledge, this is the first study to rigorously characterize the anti-hyperlipidemic activity of this SSH-based herbal formulation, including rational composition optimization, *in vitro* mechanistic profiling, and *in vivo* validation.

HFD induction remains the most widely used and physiologically relevant method for establishing murine models of diet-induced hyperlipidemia and metabolic syndrome (Munshi et al., 2014; Cheng et al., 2024; Sun et al., 2017). In this study, HFD feeding successfully induced a robust hyperlipidemic phenotype, characterized by significant elevations in serum TC, TG, and LDL-C, alongside a marked reduction in HDL-C. Concurrently, HFD-fed mice exhibited progressive weight gain and increased liver-to-body weight ratio (liver index), confirming the development of obesity-associated hepatic steatosis-a hallmark feature that enhances translational relevance to human metabolic syndrome. Intervention with SSH formulation demonstrated distinct lipid-modulating benefits in this model, significantly attenuating the elevated TC and TG levels as well as body weight and liver index. More importantly, SSH treatment not only reduced the pro-atherogenic LDL-C but also elevated the cardioprotective HDL-C levels. This “bidirectional regulation” of the lipid profile is critical, as atherogenic dyslipidemia is typified by decreased HDL-C and an increase in small, dense LDL particles (Reiner, 2017; Mu et al., 2025). Our findings therefore suggest that the SSH formulation may confer anti-atherosclerotic benefits not only by reducing overall lipid burden but also by improving lipoprotein quality, composition, and functional capacity.

Previous studies have indicated that proinflammatory cytokines are closely related to chronic diseases such as TNF-α, IL-1β and IL-6 (Ojo et al., 2022; Bin-Jumah, 2018). Consistent with this paradigm, HFD feeding triggered a pronounced hepatic inflammatory response, evidenced by significant upregulation of TNF-α and IL-6 mRNA and protein levels. Remarkably, SSH formulation treatment effectively suppressed the expression and secretion of these cytokines. These results extended the therapeutic scope of SSH beyond conventional “lipid-lowering” to encompass “anti-metabolic inflammation”-a mechanism increasingly implicated in the prevention of NAFLD progression and cardiovascular complications. We postulate that components such as Salvianolic acids from Salvia miltiorrhiza (potent anti-inflammatory and antioxidant agents) and lignans from Schisandra chinensis (activators of the Nrf2 pathway) may play a synergistic role herein, potentially by inhibiting inflammatory signaling pathways (Chun et al., 2018; Leng et al., 2017; Yan et al., 2025). This mechanism likely constitutes a key reason behind the compound’s ability to mitigate HFD-induced hepatic lipid accumulation and functional injury.

To elucidate the molecular mechanisms underlying the anti-hyperlipidemic effects of the SSH formulation, we integrated network pharmacology with experimental validation and identified seven key targets-APOB, LDLR, OLR1, PPARG, PRKCA, RXRA, and NCF1. Consistent with our *in vivo* observations, hepatic mRNA and protein expression levels of APOB, OLR1, PRKCA, RXRA, PPARG, and NCF1 were significantly upregulated in HFD-fed mice, whereas LDLR expression was markedly decreased. Critically, SSH formulation treatment fully reversed these HFD-induced dysregulations across all seven targets, indicating coordinated multi-target engagement. This coherent modulation defines a mechanistically integrated, systems-level mode of action: First, SSH-induced downregulation of APOB (essential structural component of atherogenic lipoproteins) suggests decreases in the hepatic assembly and secretion of lipid-laden particles into circulation (Dhingra and Bansal, 2006; Wang et al., 2024). Concurrently, upregulation of LDLR enhances hepatic clearance of circulating LDL-cholesterol, a classic feedback mechanism that is often impaired in hyperlipidemia (Li et al., 2021; Feng et al., 2022). Second, SSH exerted pronounced protective effects against oxidative stress and inflammation-two interdependent pathological drivers of hyperlipidemia progression and associated vascular injury. HFD-induced overexpression of OLR1 (oxidized low-density lipoprotein receptor 1) promotes endothelial dysfunction and macrophage foam cell formation by facilitating oxidized LDL uptake (Korkmaz et al., 2022; Xu et al., 2021). Furthermore, SSH-induced downregulation of PRKCA is crucial, as its activation by lipid metabolites like diacylglycerol is a well-established link between lipid overload and hepatic insulin resistance (Zhu et al., 2023). By modulating PRKCA, SSH may improve metabolic signaling (Maeda et al., 2005). SSH-induced downregulation of NCF1 (a core subunit of NADPH oxidase) directly points to a reduction in ROS generation, thereby alleviating oxidative stress (Zhang et al., 2016; Li et al., 2022). Third, our results implicated the modulation of nuclear receptor signaling. The altered expression of PPARG and its obligate heterodimer partner RXRA imply a restoration of transcriptional programs governing lipid metabolism and inflammation (Zhang et al., 2024; Lee et al., 2022). Likewise, RXRA can affect fatty acid storage, glucose metabolism, and anti-inflammatory responses (Hu et al., 2021; Yu et al., 2015; Cheng et al., 2022). Collectively, these findings suggested that the OLR1/PRKCA/ROS/NF-κB axis-a pathway implicated in oxidized lipid-triggered inflammatory responses and redox imbalance may be involved in SSH-mediated biological roles in hyperlipidemia. To verify this proposed mechanism, additional experiments were performed. We discovered that FFA caused increases of OLR1 protein expression, PRKCA protein expression and phosphorylation of NF-κB p65 as well as ROS production in HepG2 cells, which were counteracted by SSH treatment. Based on these findings, we concluded that SSH predominantly mediated its biological roles via the OLR1/PRKCA/ROS/NF-κB axis in FFA-induced HepG2 cells.

However, several limitations warrant acknowledgment and inform future research directions. Firstly, although biochemical and molecular endpoints were rigorously assessed, the current study relies primarily on targeted analyses of predefined markers. Future work should employ unbiased hepatic transcriptomics and proteomics to uncover additional regulatory nodes and pathway crosstalk. Secondly, while a whole-extract was used in this study, the exact material basis responsible for the effects and the interactions among the constituent herbs remain unclear. Future studies should integrate network pharmacology predictions with experimental validation to identify key active constituents.

## 5 Conclusion

To summarize, the therapeutic efficacy of SSH formulation is not attributed to a single target but emerges from its systemic multi-target modulation. It simultaneously regulates lipoprotein dynamics via APOB/LDLR, quenches inflammation via OLR1, PRKCA and NCF1, and controls metabolic gene expression by engaging PPARG and RXRA. This polypharmacological profile aligns with the holistic principle of Traditional Chinese Medicine.

## Data Availability

The original contributions presented in the study are included in the article/Supplementary Material, further inquiries can be directed to the corresponding authors.
