# Supplementary material for: The Schisandra-Salvia-Hawthorn herbal formulation demonstrates therapeutic efficacy in ameliorating hyperlipidemia
Source: Front Pharmacol. 2026 Oct 16;17:1893568. doi: 10.3389/fphar.2026.1893568 (PMC13719585; doi:10.3389/fphar.2026.1893568)
Supplement: Supplementary file 1 [file Table1.docx]

**Table S1** The scores of D1-D7

| Number | Scores |
| --- | --- |
| D1 | 0.49 |
| D2 | 0.55 |
| D3 | 0.58 |
| D4 | 0.58 |
| D5 | 0.67 |
| D6 | 0.61 |
| D7 | 0.56 |
